# Pathological signatures of white matter lesions in multiple sclerosis versus stroke: a synthetic MRI study

**DOI:** 10.1007/s00234-026-04002-y

**Published:** 2026-04-22

**Authors:** Evangelos Katsarogiannis, Johan Wikström, Johan Virhammar, Shala Ghaderi Berntsson, Anne-Marie Landtblom

**Affiliations:** 1 Department of Medical Sciences, Neurology, Uppsala university, Uppsala, Sweden; 2 Department of Surgical Sciences, Neuroradiology, Uppsala university, Uppsala, Sweden; 3 Department of Biomedical and Clinical Sciences, Linköping University, Linköping, Sweden; 4 Department of Medical Sciences, Neurology, Uppsala university, Uppsala, Sweden

**Keywords:** Multiple sclerosis, Stroke, Quantitative synthetic MRI, White matter lesions

## Abstract

**Purpose:**

To evaluate whether quantitative synthetic MRI (SyMRI) parameters can differentiate non-specific white matter lesions (nsWMLs) in patients with multiple sclerosis (MS) and ischemic stroke, and to assess differences in normal-appearing white matter (NAWM) between these groups.

**Methods:**

Thirty MS patients and nineteen ischemic stroke patients underwent standardized MRI including SyMRI. Three lesion categories were analyzed: typical MS lesions (MSL), non-specific lesions in MS (nsWML-MS), and non-specific lesions in stroke (nsWML-S). SyMRI-derived parameters (R1, R2, proton density, and myelin content) were extracted from each region of interest (ROI), and one ROI was placed in NAWM per patient. Group differences were evaluated using non-parametric tests. Logistic regression models, both unadjusted and age-adjusted, assessed predictors of MS diagnosis.

**Results:**

Typical MS lesions showed lower myelin content and R1 and higher proton density than nsWML-MS (all *p* < 0.0001). Compared with nsWML-S, nsWML-MS demonstrated lower myelin content and higher proton density (*p* < 0.05), while R1 and R2 values did not differ. NAWM differences between MS and stroke emerged only after age adjustment. Age alone discriminated MS from stroke (AUC 0.83), with modest improvement when NAWM measures were added (AUC 0.86).

**Conclusion:**

SyMRI captures both lesion-specific and diffuse NAWM differences between MS and stroke. Age strongly influences quantitative white matter measures, and adjusting for age reveals subtle NAWM pathology in MS. SyMRI may support differential diagnosis in patients with ambiguous white matter lesions.

**Supplementary Information:**

The online version contains supplementary material available at 10.1007/s00234-026-04002-y.

## Introduction

Multiple sclerosis (MS) and stroke are two major neurological disorders contributing significantly to disability worldwide. Although their clinical and epidemiological profiles differ, overlap exists: MS patients have a 2.55-fold higher relative risk of developing stroke [1].

White matter lesions (WMLs) are a common radiological finding in both MS and stroke. Non-specific white matter lesions (nsWMLs) are defined as T2- or FLAIR-hyperintense regions without clear morphological features that clarify their etiology. They are frequently observed with aging and/or vascular risk factors, have no typical location, and can be observed in periventricular, subcortical as well as deep white matter regions [2, 3]. The presence of nsWMLs complicates radiological diagnosis of MS as their ambiguous appearance makes it difficult to distinguish them from ischemic lesions, particularly in older patients and/or those with vascular comorbidities [4–6]. A predominance of non-perivenular lesions, especially subcortical, may signal alternative or comorbid vascular pathology [7].

nsWMLs may reflect distinct pathogenetic backgrounds in MS and stroke respectively, but it is not excluded that the origin may have common features. Interestingly, recent research reveals that in MS, impaired perfusion, mitochondrial dysfunction, and inflammation create a self-reinforcing hypoxia–inflammation cycle that drives demyelination and disability [8, 9]. And in stroke, a secondary demyelination is increasingly recognized as a key driver of long-term deficits. This involves collagen-mediated inhibition of remyelination [10], astrocytic lipocalin-2 signaling [11], and persistent myelin deficits that exacerbate neuronal loss [12]. Thus, both disorders show convergent myelin vulnerability despite different initiating mechanisms.

Synthetic MRI (SyMRI) is a quantitative imaging technique that, from one multidynamic multiecho sequence, generates T1-, T2-, and PD-weighted images, volumetric segmentations, and quantitative maps of R1, R2, PD, and myelin content (MyC) [13–15]. In MS imaging, SyMRI matches conventional MRI lesion detection while reducing scan time. Previous work comparing synthetic and conventional MRI in patients with MS demonstrated comparable diagnostic image quality and no significant differences in cortical lesion counts or lesion volumes between the two approaches. Additionally, synthetic MRI enables the generation of multiple contrast-weighted images from a single quantitative acquisition, reducing total scan time by approximately 50% while maintaining diagnostic utility [16]. Subtraction mapping may further enhance the sensitivity for detecting new lesions [17]. Paramagnetic rim lesions identified with SyMRI may reflect diffuse periplaque damage and ongoing silent progression [18], and combined PET–SyMRI studies have reported associations between microglial activation, myelin loss, and clinical decline [19].

In diagnostic imaging of stroke, SyMRI enables estimation of relaxation times with minimal discrepancy compared to conventional MRI [20]. Synthetic FLAIR performs comparably to standard FLAIR in early ischemia, with reduced scan time [21]. Quantitative values (R1, R2, PD) can possibly distinguish acute from chronic ischemia [22] and stratify stroke severity [23]. Moreover, SyMRI-derived total myelin volume can be linked to 3-month functional outcomes [24]. One early SyMRI study assessed a small group of patients with MS, stroke and borderline cases [14].

The objectives of the study were to: first, assess the value of quantitative parameters derived from SyMRI for differentiating non-specific white matter lesions in two different patient cohorts, one with MS and one with ischemic stroke; and second, to investigate whether this method can differentiate between typical and non-specific white matter lesions in the MS cohort.

## Methods

### Study population

Initially, 32 patients with ischemic stroke aged 60 years or younger were screened for participation. Of these, 13 were excluded because they had only the index ischemic stroke lesion without additional white matter lesions on MRI, or had lesions that did not meet the inclusion criteria, resulting in a final sample of 19 stroke patients. The stroke cohort consisted of patients examined in a non-acute phase after the index ischemic stroke (median time from stroke to MRI 507 days; IQR 339–849 days). In addition, 30 patients with clinically confirmed multiple sclerosis (MS) were included, yielding a total study population of 49 participants.

All participants underwent a single MRI examination at Uppsala University Hospital between 2021 and 2024, utilizing a standardized imaging protocol. Inclusion criteria for all patients included, being ≥ 18 years of age and availability of a complete MRI imaging including fluid-attenuated inversion recovery (FLAIR) and synthetic MRI sequences. For the MS cohort, demographic data, expanded disability status scale scores (EDSS), and data on disease duration, time since last relapse, MS subtypes, and distribution of disease-modifying therapies (DMTs) were collected. EDSS was presented as median (interquartile range, IQR) (Table 1).

**Table 1:** Clinical characteristics of patients with multiple sclerosis (MS)

Clinical characteristics MS patients	
Number of MS patients, n	30
Age, mean ± SD (years) Male sex	47.3 ± 7.53 5 (17%)
EDSS, median (IQR)	2.0 (1.5–3.0)
Time since MS onset, mean ± SD (months)	174.3 ± 88.3
Time since last relapse, mean ± SD (months)	74.8 ± 35.4
MS Type (n/%)	
RRMS	27 (90.0%)
SPMS	3 (10.0%)
Type of DMT (n/%)	
Rituximab	13 (43.0%)
HSCT	8 (26.7%)
Dimethyl fumarate	3 (10.0%)
None	2 (6.7%)
Cladribine	1 (3.3%)
Alemtuzumab	1 (3.3%)
Fingolimod	1 (3.3%)
INF	1 (3.3%)

As for the patients with ischemic stroke, data on demographic parameters, vascular risk factors, cardiac comorbidities, and stroke subtypes were retrieved from medical records (Table 2).

**Table 2:** Clinical characteristics of patients with ischemic stroke

Clinical characteristics Stroke patients	
Number of patients, n	19
Age, y (mean ± SD)	54.5 ± 4.4
Male sex	13 (68%)
BMI > 25, n (%)	3 (15.8%)
Hypertension, n (%)	13 (68.4%)
Diabetes, n (%)	4 (21.1%)
Dyslipidemia, n (%)	6 (31.6%)
Atrial Fibrillation, n (%)	2 (10.5%)
Smoking, n (%)	2 (10.5%)
Chronic heart failure, n (%)	1 (5.3%)
Chronic kidney disease, n (%)	1 (5.3%)
Patent foramen ovale, n (%)	4 (21.1%)
Cryptogenic stroke, n (%)	1 (5.3%)
Stroke subtype	
Large-artery atherosclerosis, n (%)	5 (26.3%)
Cardioembolic stroke, n (%)	1 (5.3%)
Small-vessel occlusion, n (%)	12 (63.2%)
Other cause, n (%)	1 (5.3%)

### MRI acquisition

All MRI scans were performed on a 3T scanner (Achieva dStream, Philips Medical Systems, Best, The Netherlands) equipped with a 32-channel head coil. The imaging protocol included a three-dimensional FLAIR sequence and a multi-dynamic multi-echo (MDME) sequence optimized for synthetic MRI acquisition. In the stroke cohort, diffusion-weighted imaging (DWI) was additionally available and was reviewed for lesion characterization and exclusion of acute ischemic lesions.

Specific sequence parameters were as follows: FLAIR (TR = [4800] ms, TE = [304] ms, TI = [1650] ms, slice thickness = [1.1] mm) and MDME (TR = [4688] ms, TE = [12.5] ms, slice thickness = [4] mm). The MDME sequence enabled the generation of quantitative maps of R1, R2 relaxation rates, proton density (PD), and myelin content using the SyMRI software versions (7.1, 2017; 7.3, 2018; 8.0, 2019;11.0.7, 2019; 12.1.11, 2024) Synthetic MR AB, Linköping, Sweden).

In addition to quantitative parameter maps, SyMRI reconstructs synthetic contrast-weighted images (including T1-, T2-, and FLAIR-weighted images) derived directly from the same quantitative dataset, ensuring inherent spatial correspondence between the anatomical images and the quantitative maps.

### Lesion selection and roi placement

Criteria for typical MS lesions included periventricular or corpus callosum location, ovoid shape, perpendicular orientation of the long axis towards the adjacent lateral ventricle, and size > 5 mm.

For each patient, representative non-specific white matter lesions were initially identified on the conventional FLAIR sequence, which served as the anatomical reference for lesion detection. Non-specific lesions were defined as FLAIR-hyperintense lesions lacking typical morphological features of MS lesions and meeting the predefined inclusion criteria: lesion size > 5 mm in maximum diameter, located at least 10 mm from the ventricular system and 5 mm from the cortical surface (outer edge of FLAIR hyperintensity), absence of contrast enhancement, and lesions not appearing hypointense on T1-weighted sequences.

In the stroke cohort, diffusion-weighted imaging (DWI) was reviewed to confirm the absence of acute ischemic lesions. Lesions demonstrating diffusion restriction were therefore not included in the analysis. In addition, lesions with imaging characteristics typical of infarction, such as those corresponding to a defined arterial vascular territory or demonstrating cerebrospinal fluid–like signal compatible with chronic lacunar infarction, were excluded.

After lesion identification on conventional FLAIR images, the corresponding lesions were visually matched to the synthetic FLAIR images generated within the SyMRI software environment. Regions of interest (ROIs) were subsequently delineated on the synthetic FLAIR images. Because the synthetic images are reconstructed directly from the same quantitative dataset, they are inherently spatially aligned with the quantitative maps, and no additional anatomical co-registration step was required. Once the ROIs were placed, the SyMRI software automatically extracted the corresponding quantitative parameters (R1, R2, proton density, and myelin content) from the underlying maps.

During ROI placement, care was taken to maintain a sufficient margin between the ROI borders and the lesion edges as well as adjacent anatomical boundaries to minimize partial volume effects. Only lesions providing adequate surrounding white matter space for reliable ROI placement were included in the analysis.

In addition to lesion analysis, one ROI was placed in normal-appearing white matter (NAWM) for each of the 30 MS and 19 stroke patients. NAWM was visually identified on conventional and SyMRI-generated synthetic FLAIR images, as white matter regions without visible abnormalities. ROIs were placed in visually normal regions at a sufficient distance from focal lesions to minimize partial volume effects.

### Quantitative MRI Analysis

Quantitative evaluation of the selected lesions was conducted using the SyMRI post-processing software. For each ROI, R1, R2, proton density, and myelin content (MyC) were extracted. In addition to lesion analysis, a ROI was also placed within normal-appearing white matter (NAWM) to obtain reference measurements for comparison. Lesion selection was initially performed by a first rater who was not blinded to the patient group (MS or stroke). A second rater, an experienced neuroradiologist, reviewed all lesions under blinded conditions; however, full blinding could not always be ensured when overt radiological features suggested the underlying diagnosis.

### Statistics

For all analyses, the mean value for the lesions was calculated at the patient level. Comparisons between typical MS lesions (MSL) and non-specific lesions in MS patients (nsWML-MS) were performed using paired Wilcoxon signed-rank tests, while differences between nsWML-MS and non-specific lesions in stroke patients (nsWML-S) were assessed using Mann–Whitney U tests. Logistic regression models, both unadjusted and adjusted for age, were applied to MyC, PD, R1, and R2 values to predict MS status, with results reported as odds ratios (OR) and 95% confidence intervals (CI). Receiver operating characteristic (ROC) curves were generated to evaluate discriminatory ability. Comparisons of normal-appearing white matter (NAWM) between MS and stroke groups were also conducted using Mann–Whitney U tests and age-adjusted logistic regression.

## Results

The demographic and clinical characteristics of the MS and stroke cohorts are summarized in Tables 1 and 2.

**Fig. 1:**
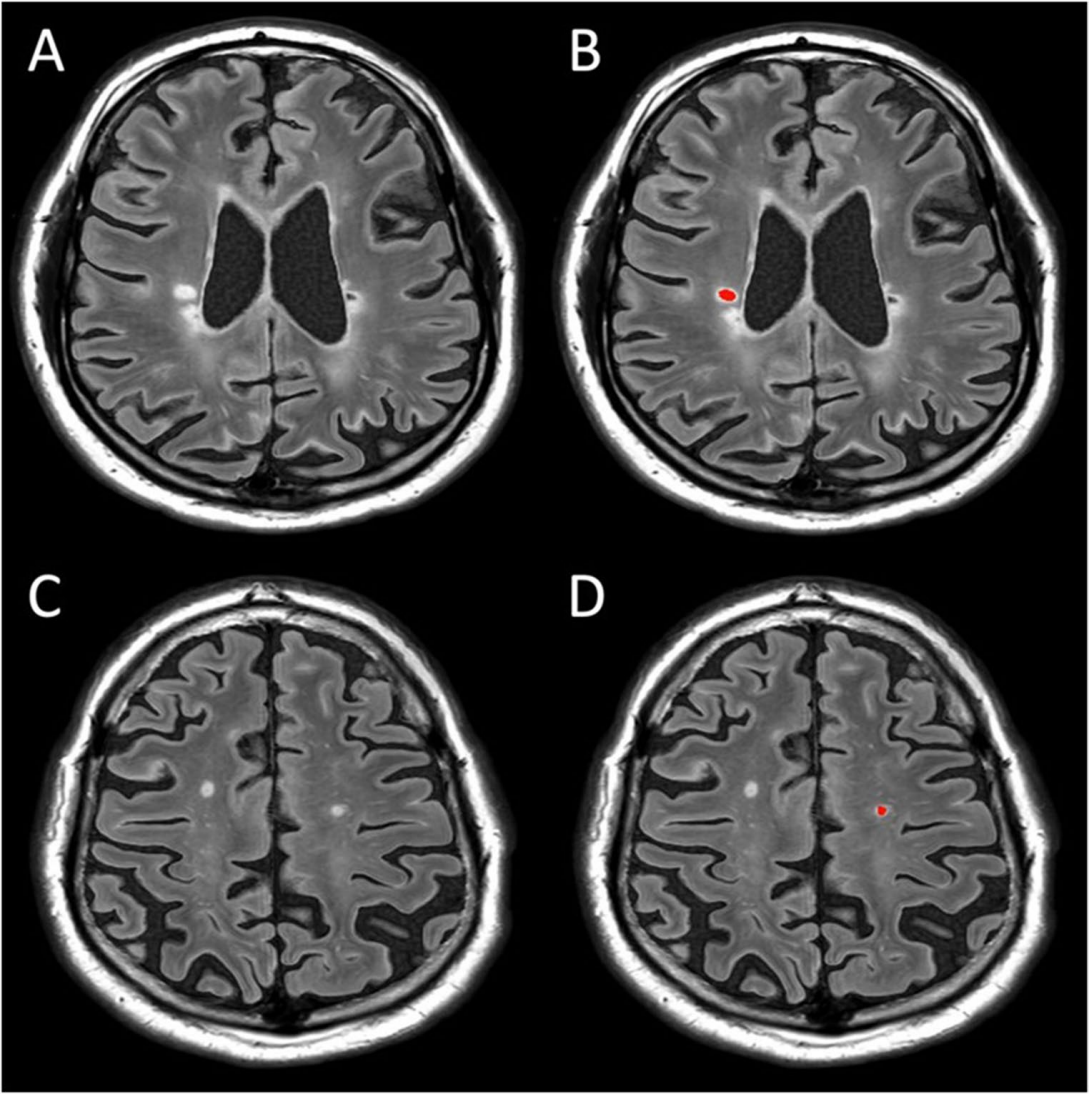
Examples of *before* and *after* ROI placement (in red) across lesion types in multiple sclerosis (MS) on SyMRI-generated synthetic FLAIR images (A–B) Typical MS lesion (C–D) Non-specific white-matter lesion (nsWML-MS) in an MS patient Lesions are grouped by patient diagnosis for illustration; selection was performed blinded to diagnosis

**Fig. 2:**
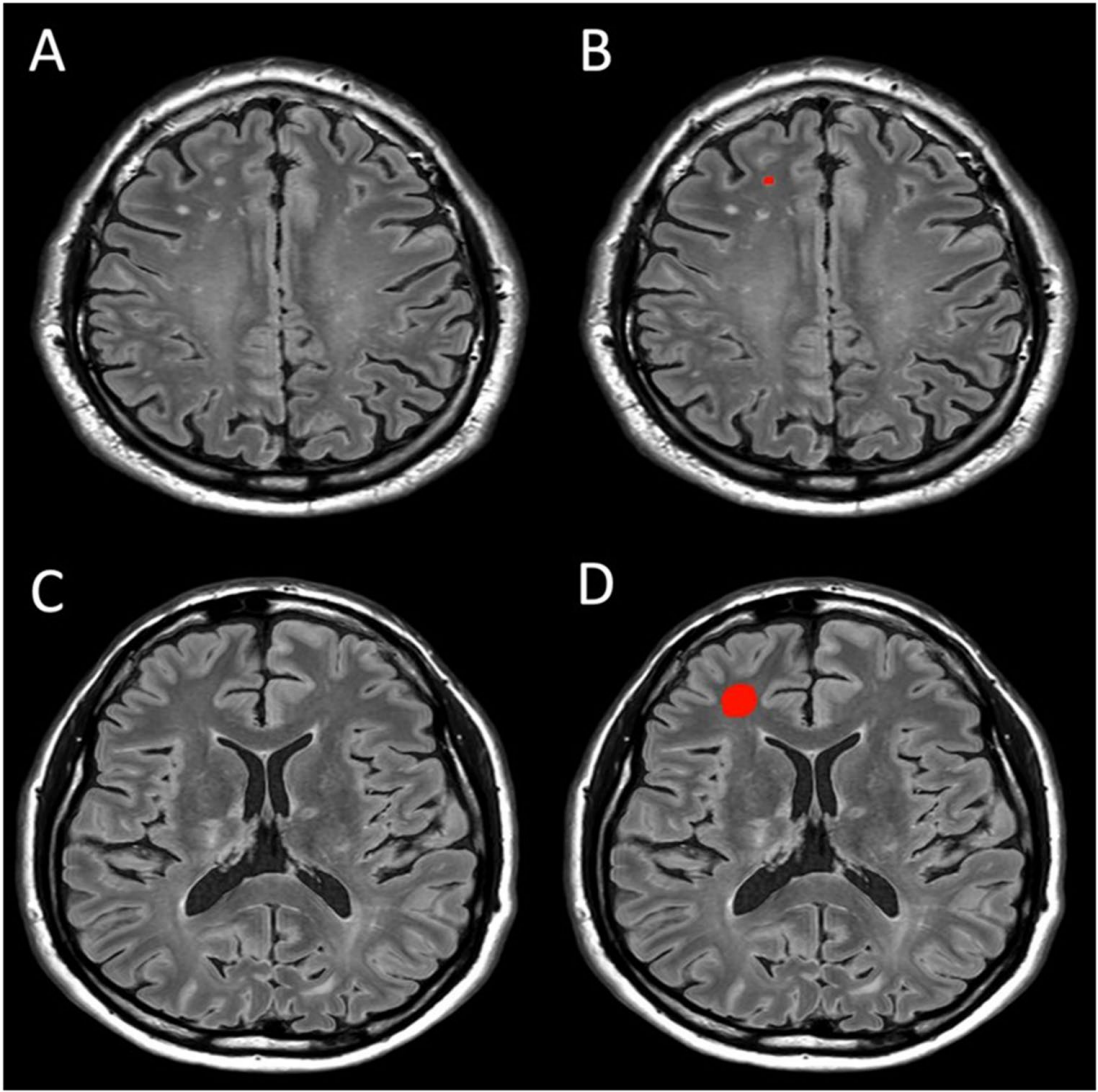
Examples of *before* and *after* ROI placement (in red) in stroke patients and NAWM on SyMRI-generated synthetic FLAIR images (A–B) Non-specific white-matter lesion (nsWML-S) in a stroke patient (C–D) Normal-appearing white matter (NAWM) in the same subject Lesions are grouped by patient diagnosis for illustration; selection was performed blinded to diagnosis

In total, 157 white matter lesions were analyzed: 61 typical MS lesions (MSL), 48 non-specific white matter lesions in MS patients (nsWML-MS), and 48 non-specific white matter lesions in stroke patients (nsWML-S). Online Resource Supplementary Table 1 shows the distribution of lesions among patients (Figs 1 and 2).

### Comparison of typical MS lesions (MSL) and Non-Specific lesions in MS patients (nsWML-MS)

Of the 30 MS patients, 24 had at least one supratentorial MSL that met the size criterion (> 5 mm). These 24 patients represented a total of 61 MSLs and 40 nsWML-MSs.

**Fig. 3:**
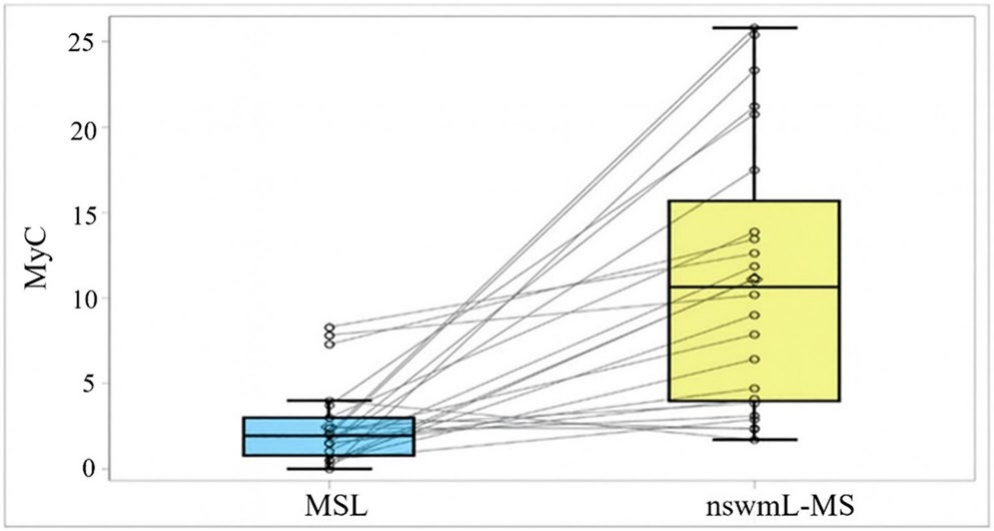
Boxplots show myelin content (MyC) in typical (MSL) and non-specific lesions (nsWML-MS) in 24 MS patients (Wilcoxon *p* < 0.0001)

Paired analyses within 24 MS patients (61 MSL and 40 nsWML-MS) showed that the median myelin content (MyC) was markedly lower in MSL compared to nsWML-MS (1.98 vs. 10.63, *p* < 0.0001), (Fig. 3). Proton density (PD) was significantly higher in MSL (89.93 vs. 80.63, *p* < 0.0001), while R1 and R2 relaxation rates were significantly reduced (both *p* < 0.0001), (Table 3).

**Table 3:** Descriptive statistics and Wilcoxon signed-rank test results for non-specific white matter lesions in MS patients (nsWML-MS) and typical MS lesions (MSL) in MS patients (*n* = 24)

SyMRI Parameters	nsWML-MS Median (IQR)	MSL Median (IQR)	Mean ± SD (nsWML-MS)	Mean ± SD (MSL)	*p*-value
Myelin conten (MyC)	10.63 (3.96–15.68)	1.98 (0.79–3.00)	11.10 ± 7.73	2.47 ± 2.32	< 0.0001
Proton density (PD)	80.63 (74.55–85.40)	89.93 (87.18–91.73)	79.94 ± 6.39	89.30 ± 4.88	< 0.0001
R1	0.92 (0.82–0.99)	0.74 (0.71–0.77)	0.92 ± 0.11	0.75 ± 0.08	< 0.0001
R2	10.37 (9.91–11.02)	9.20 (8.30–9.42)	10.46 ± 1.11	8.96 ± 0.83	< 0.0001

### Comparison of Non-Specific lesions in MS (nsWML-MS) and stroke (nsWML-S) patients

A total of 48 nsWML-MS and 48 nsWML-S lesions were included. nsWML-MS had significantly lower MyC than nsWML-S (12.23 vs. 16.40, *p* = 0.0489), and significantly higher PD (79.11 vs. 74.50, *p* = 0.0380). In contrast, differences in R1 (*p* = 0.1603) and R2 (*p* = 0.2520) between the two groups did not reach statistical significance (Table 4).

**Table 4:** Comparison between non-specific white matter lesions in MS (nsWML-MS) and stroke (nsWML-S). Values are shown as Median (IQR) and Min–Max

SyMRI Parameters	nsWML-MS Median (IQR)	nsWML-MS Min–Max	nsWML-S Median (IQR)	nsWML-S Min–Max	*P*-value
MyC (ml)	12.23 (4.70–17.50)	1.70–25.80	16.40 (11.80–20.95)	4.85–29.95	0.0489
PD	79.11 (74.20–83.80)	68.40–90.50	74.50 (71.65–76.20)	66.25–86.60	0.0380
R1	0.95 (0.84–0.99)	0.74–1.15	0.97 (0.87–1.04)	0.83–1.16	0.1603
R2	10.40 (9.91–11.10)	8.38–12.99	10.91 (9.81–11.51)	9.38–12.78	0.2520

### Logistic regression analysis

Age-adjusted logistic regression models were fitted using patient-level mean values of MyC, PD, R1, and R2 from nsWML-MS and nsWML-S lesions. In unadjusted analyses, higher PD and lower MyC were associated with increased odds of MS (PD: OR 1.13, 95% CI 1.02–1.28, *p* = 0.020; MyC: OR 0.91, 95% CI 0.83–1.00, *p* = 0.038). However, after adjusting for age, these associations were no longer statistically significant. Receiver operating characteristic (ROC) analysis indicated that age alone discriminated MS from stroke with an AUC of 0.83 (95% CI 0.71–0.95). Adding SyMRI parameters to age did not substantially improve discrimination (Table 5).

**Table 5:** Logistic regression models predicting MS status based on mean values of non-specific white matter lesions in MS (nsWML-MS) and stroke (nsWML-S)

SyMRI parameters	Unadjusted OR (95% CI), *p*	Age-adjusted OR (95% CI), *p*	AUC (95% CI)	AUC/ Age (95% CI)
MyC	0.91 (0.83–1.00), *p* = 0.038	0.96 (0.87–1.06), *p* = 0.452	0.67 (0.52–0.83)	0.84 (0.73–0.96)
PD	1.13 (1.02–1.28), *p* = 0.020	1.06 (0.93–1.21), *p* = 0.371	0.68 (0.53–0.84)	0.85 (0.73–0.97)
R1	0.64 (0.33–1.14), *p* = 0.131	0.82 (0.40–1.67), *p* = 0.578	0.62 (0.46–0.78)	0.83 (0.71–0.95)
R2	0.72 (0.40–1.26), *p* = 0.252	0.72 (0.36–1.42), *p* = 0.343	0.60 (0.43–0.77)	0.84 (0.73–0.96)
Age	0.85 (0.75–0.93), *p* < 0.001	-	0.83 (0.71–0.95)	-

### Correlation analysis

Pearson’s correlation analysis revealed negative correlations between MyC and PD (*r* = − 0.97 for nsWML-MS, *r* = − 0.96 for nsWML-S, both *p* < 0.0001), suggesting redundancy between these measures. Positive correlations were also observed between MyC and R1, and negative between PD and R2. In nsWML-MS, MyC showed a moderate positive correlation with age (*r* = 0.47, *p* = 0.008), whereas in nsWML-S, no significant correlations with age were observed (Online Resource Supplementary Tables 2 and 3).

### Comparison of normal-appearing white matter (NAWM)

**Fig. 4:**
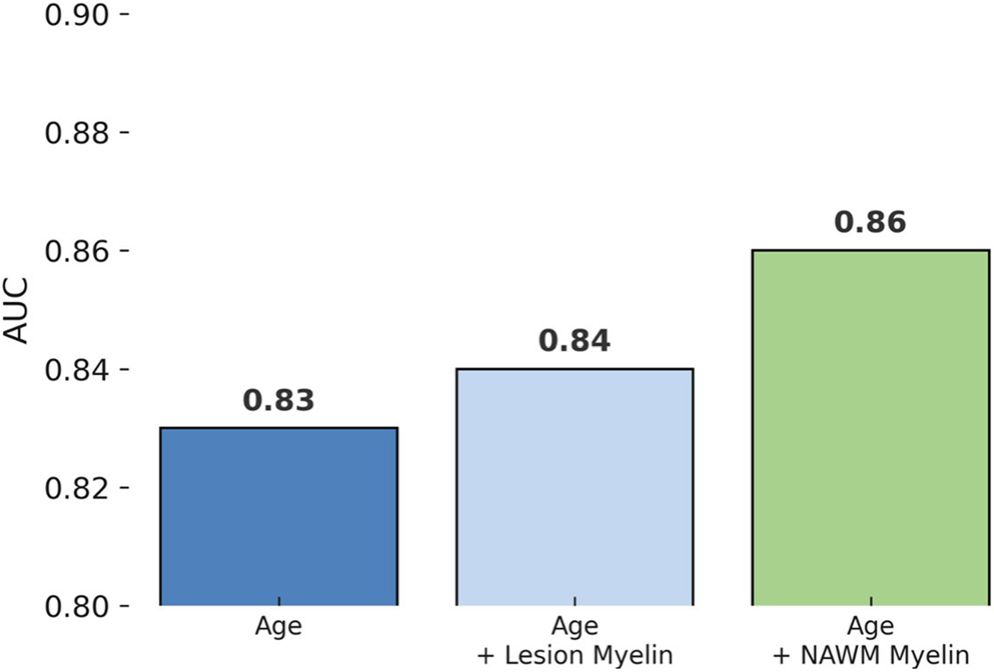
Diagnostic performance (ROC analysis) for differentiating multiple sclerosis (MS) from stroke. AUC values for models using age alone (0.83), age + lesion myelin (0.84), and age + NAWM myelin (0.86). The y-axis is restricted to 0.80–0.90 to enhance visual clarity

NAWM variables did not differ significantly between MS and stroke patients. MyC was marginally lower and PD marginally higher in the MS group, and R1 showed a trend toward reduction in MS compared to stroke group (*p* = 0.068) (Online Resource Supplementary Table 4). Logistic regression on NAWM parameters indicated that, after age adjustment, lower MyC (OR 0.76, 95% CI 0.56–0.98, *p* = 0.031), higher PD (OR 1.52, 95% CI 1.04–2.40, *p* = 0.032), and reduced R1 (OR 0.25, 95% CI 0.06–0.70, *p* = 0.007) were significantly associated with MS. ROC analysis showed that age alone achieved an AUC of 0.83, while the addition of NAWM parameters slightly improved classification accuracy (up to AUC 0.86) (Table 6). As shown in Fig. 4, age alone achieved an AUC of 0.83. When myelin content from lesions was added to the model, the AUC increased modestly to 0.84, and inclusion of myelin content from NAWM reached 0.86, indicating a limited incremental contribution of quantitative myelin measures beyond age.

**Table 6:** Logistic regression models predicting MS status based on normal-appearing white matter (NAWM) parameters

Parameter	Unadjusted OR (95% CI), *p*	Age-adjusted OR (95% CI), *p*	AUC (95% CI)	AUC incl. Age (95% CI)
MyC	0.87 (0.70–1.06), *p* = 0.161	0.76 (0.56–0.98), *p* = 0.031	0.65 (0.49–0.81)	0.86 (0.75–0.97)
PD	1.24 (0.92–1.72), *p* = 0.163	1.52 (1.04–2.40), *p* = 0.032	0.65 (0.48–0.81)	0.86 (0.75–0.97)
R1	0.42 (0.16–0.96), *p* = 0.038	0.25 (0.06–0.70), *p* = 0.007	0.66 (0.50–0.82)	0.86 (0.75–0.97)
R2	0.63 (0.27–1.39), *p* = 0.254	0.42 (0.14–1.07), *p* = 0.069	0.59 (0.42–0.76)	0.86 (0.75–0.98)
Age	0.85 (0.75–0.93), *p* < 0.001	-	0.83 (0.71–0.95)	-

## Discussion

Within the MS group, typical MS lesions (MSL) differed significantly from non-specific lesions (nsWML-MS). Typical MS lesions showed lower MyC and R1 values, together with higher PD, consistent with more advanced demyelination and tissue loss. In contrast, nsWML-MS retained higher myelin content and relaxation values, closer to normal compared with MSL, suggesting milder demyelination or a different injury pathway. These findings support the view that not all MS-associated white matter lesions are equal and that SyMRI can detect gradations of pathology that conventional imaging cannot capture.

When comparing nsWML-MS with nsWML-S, we observed higher MyC and lower PD in stroke patients. These findings may be compatible with differences in the underlying tissue processes of MS and stroke: in MS, involving inflammation-related demyelination, and in stroke, reflecting ischemia-related secondary myelin injury [12].

Similarly, NAWM values showed only subtle differences between MS and stroke patients, with MS tending toward lower myelin content (MyC) and higher proton density (PD). While these differences did not reach significance in direct group comparisons, age-adjusted regression analyses indicated that lower MyC and higher PD were independently associated with MS diagnosis. Previous studies have reported reduced NAWM myelin fractions in MS patients compared to healthy controls. These reductions are correlated to both physical and cognitive disability, underscoring the clinical importance of diffuse white matter damage [25]. Additional studies have found reduced myelin volume fraction (MVF) and altered relaxometry metrics (R1, R2, PD) in NAWM compared to controls, while further work has demonstrated that SyMRI-derived MVF in NAWM correlates with disease duration, highlighting the method’s sensitivity to disease burden [26, 27]. These findings align with longitudinal magnetization transfer studies showing that microstructural alterations in NAWM can precede focal lesion formation, indicating a pre-lesional stage of tissue vulnerability [28]. This suggests that diffuse NAWM abnormalities captured by quantitative MRI may reflect early pathological changes preceding overt demyelination.

In stroke, NAWM undergoes early microstructural alterations, but the underlying mechanism appears distinct. Previous studies indicate that NAWM regions, that later evolved into white matter hyperintensities, already exhibit reduced white-matter–like signals and elevated fluid- and gray-matter–like components early-on, suggesting tissue vulnerability before lesion formation [29]. A recent study has shown that even beyond visible leukoaraiosis, NAWM exhibits subtle and diffuse FLAIR signal increases, correlating with overall leukoaraiosis burden and age [30].

Our results extend these observations by suggesting that NAWM metrics might provide additional discriminative power between MS and stroke compared to lesion-based comparisons alone. The lower myelin content and higher PD seen in MS NAWM may reflect widespread inflammatory demyelination, whereas the minimal signal changes observed in stroke-related NAWM may be related to diffuse vascular injury. This distinction suggests a potential role for SyMRI in disentangling the different biological processes underlying NAWM changes, improving diagnostic accuracy when conventional MRI findings are inconclusive.

As expected, age emerged as a strong discriminator in the logistic regression analysis. This finding partly reflects the study design, as patients with stroke were older than those with MS. However, it also mirrors the well-established biological context: vascular-related white matter changes accumulate with advanced age, whereas MS typically presents earlier in life. Importantly, this underscores that SyMRI parameters cannot be interpreted in isolation but need to be evaluated together with demographic and clinical data to improve diagnostic accuracy. NAWM parameters became significant only after age adjustment, suggesting that age exerts a strong influence on white matter tissue properties. Controlling for this effect allowed disease-related differences in MS NAWM to emerge more clearly, implying that a quantitative MRI of NAWM may detect indications of pathology once age-related variance is accounted for.

Several advanced imaging techniques have been explored to address the diagnostic challenge of distinguishing MS lesions from non-specific white matter lesions. For instance, biomarkers such as the central vein sign and percentage of perivenous white matter lesions (% PVWML) obtained from 7T SWI/FLAIR imaging can help differentiate MS lesions from other WMLs [31]. However, their diagnostic accuracy decreases when non-specific white matter lesions exhibit imaging characteristics similar to MS lesions, such as the presence of a central vein or perivenous distribution, creating challenges to differentiation [32, 33]. Functional BOLD (blood-oxygen-level-dependent) imaging has shown outward signal reductions in MS lesions compared to nsWMLs [34], and convolutional neural network models have achieved up to 78% accuracy in separating MS from non-specific WMLs based on MRI data [35]. Our findings indicate that SyMRI may complement these more specialized methods by providing both quantitative information together with lesion-specific and diffuse NAWM changes in a single, clinically feasible scan, potentially enhancing conventional MRI imaging by providing diagnostic value.

Limitations on our study, include the modest sample size, a single-center design, lack of histopathological validation, and absence of a healthy control group for comparison. Lesion selection was performed by one rater and reviewed by a blinded neuroradiologist to ensure consistency. Nevertheless, inter-rater reproducibility was not formally quantified, which may introduce minor subjectivity. Although the median values of SyMRI parameters differed significantly between groups, there was considerable overlap in the distributions, with individual lesions often falling within similar ranges for MS and stroke. This suggests that SyMRI metrics alone are unlikely to provide a definitive diagnostic classification, and their greatest value may lie in combination with clinical and demographic factors.

## Conclusions

SyMRI-derived quantitative parameters demonstrated differences between non-specific white matter lesions in patients with multiple sclerosis and ischemic stroke. In addition, modest differences were observed in normal-appearing white matter (NAWM) between the groups, suggesting that diffuse white matter alterations may also contribute to the distinction between the two conditions. However, considerable overlap between groups was present, and the discriminatory performance remained moderate. These findings indicate that SyMRI may provide additional quantitative information when evaluating ambiguous white matter lesions, although further validation in larger cohorts is required before clinical implementation. 

## Supplementary Information

Below is the link to the electronic supplementary material.

Supplementary Material 1 (DOCX 17.7 KB)

## Data Availability

Data are available from the corresponding author on reasonable request.
