# Supplementary material for: Pathological signatures of white matter lesions in multiple sclerosis versus stroke: a synthetic MRI study
Source: Neuroradiology. 2026 Apr 22;68(6):1631–9. doi: 10.1007/s00234-026-04002-y (PMC13323269; doi:10.1007/s00234-026-04002-y)
Supplement: Supplementary file 1 — Supplementary Material 1 (DOCX 17.7 KB) [file 234_2026_4002_MOESM1_ESM.docx]

**Supplementary tables**

# **Online Resource.** **Supplementary table 1.** Number of lesions per patient

Distribution of typical MS lesions (MSL), non-specific lesions in MS patients (nsWML-MS), and non-specific lesions in stroke patients (nsWML-S).

|  | Number of patients | | |
| --- | --- | --- | --- |
| Number of Lesions | MSL (n) | nsWML-MS (n) | nsWML-S (n) |
| 1 | 3 | 14 | 5 |
| 2 | 11 | 14 | 9 |
| 3 | 5 | 2 | 2 |
| 4 | 4 | - | 1 |
| 5 | 1 | - | - |
| 7 | - | - | 1 |
| 8 | - | - | 1 |
| Total Patients | 24 | 30 | 19 |

**Online Resource.** **Supplementary table 2.** Pearson correlations between SyMRI parameters and age in non-specific white matter lesions in MS (nsWML-MS) and stroke (nsWML-S).

| Variable 1 | Variable 2 | r | p-value |
| --- | --- | --- | --- |
| MyC (nsWML-MS) | PD (nsWML-MS) | -0.97 | <0.0001 |
| MyC (nsWML-MS) | R1 (nsWML-MS) | 0.84 | <0.0001 |
| MyC (nsWML-MS) | R2 (nsWML-MS) | 0.69 | <0.0001 |
| PD (nsWML-MS) | R1 (nsWML-MS) | -0.81 | <0.0001 |
| PD (nsWML-MS) | R2 (nsWML-MS) | -0.64 | <0.0001 |
| MyC (nsWML-MS) | Age | 0.47 | 0.008 |
| MyC (nsWML-S) | PD (nsWML-S) | -0.96 | <0.0001 |
| MyC (nsWML-S) | R1 (nsWML-S) | 0.83 | <0.0001 |
| MyC (nsWML-S) | R2 (nsWML-S) | 0.74 | <0.0001 |
| PD (nsWML-S) | R1 (nsWML-S) | -0.81 | <0.0001 |
| PD (nsWML-S) | R2 (nsWML-S) | -0.72 | <0.0001 |
| MyC (nsWML-S) | Age | 0.17 | 0.361 |

**Online Resource.** **Supplementary table 3.** Pearson correlations between SyMRI parameters and age in normal-appearing white matter (NAWM) of MS and stroke patients.

| Variable 1 | Variable 2 | r | p-value |
| --- | --- | --- | --- |
| MyC (MS) | PD (MS) | -0.95 | <0.0001 |
| MyC (MS) | R1 (MS) | 0.88 | <0.0001 |
| MyC (MS) | R2 (MS) | 0.74 | <0.0001 |
| PD (MS) | R1 (MS) | -0.89 | <0.0001 |
| PD (MS) | R2 (MS) | -0.75 | <0.0001 |
| MyC (MS) | Age | 0.33 | 0.120 |
| MyC (Stroke) | PD (Stroke) | -0.94 | <0.0001 |
| MyC (Stroke) | R1 (Stroke) | 0.91 | <0.0001 |
| MyC (Stroke) | R2 (Stroke) | 0.83 | <0.0001 |
| PD (Stroke) | R1 (Stroke) | -0.92 | <0.0001 |
| PD (Stroke) | R2 (Stroke) | -0.84 | <0.0001 |
| MyC (Stroke) | Age | 0.05 | 0.792 |

**Online Resource.** **Supplementary Table 4**. Comparison of normal-appearing white matter (NAWM) between MS and stroke patients.

| SyMRI  Parameters | MS Median (IQR) | MS Min–Max | Stroke Median (IQR) | Stroke  Min–Max | P-value |
| --- | --- | --- | --- | --- | --- |
| MyC | 36.35 (35.60–38.20) | 30.90–44.40 | 38.00 (36.10–40.40) | 31.60–43.20 | 0.0930 |
| PD | 61.85 (60.60–62.30) | 56.50–65.40 | 60.70 (59.20–61.90) | 57.30–64.90 | 0.0968 |
| R1 | 1.42 (1.37–1.46) | 1.29–1.62 | 1.45 (1.41–1.52) | 1.31–1.57 | 0.0680 |
| R2 | 15.61 (14.98–16.13) | 14.13–16.54 | 15.73 (15.22–16.46) | 14.11–17.26 | 0.3053 |
